# Harmonization of Rapid Evaporative Ionization Mass Spectrometry Workflows across Four Sites and Testing Using Reference Material and Local Food-Grade Meats

**DOI:** 10.3390/metabo12111130

**Published:** 2022-11-17

**Authors:** Martin Kaufmann, Pierre-Maxence Vaysse, Adele Savage, Ala Amgheib, András Marton, Eftychios Manoli, Gabor Fichtinger, Steven D. Pringle, John F. Rudan, Ron M. A. Heeren, Zoltán Takáts, Júlia Balog, Tiffany Porta Siegel

**Affiliations:** 1 Department of Surgery, Queen’s University, Kingston, ON K7L 2V7, Canada; 2 Department of Biomedical and Molecular Sciences, Queen’s University, Kingston, ON K7L 3N6, Canada; 3 Maastricht MultiModal Molecular Imaging (M4i) Institute, Division of Imaging Mass Spectrometry, Maastricht University, 6229 ER Maastricht, The Netherlands; 4 Department of Surgery, Maastricht University Medical Center + (MUMC+), 6229 HX Maastricht, The Netherlands; 5 Department of Otorhinolaryngology, Head & Neck Surgery, MUMC+, 6229 HX Maastricht, The Netherlands; 6 Division of Computational and Systems Medicine, Department of Surgery and Cancer, Imperial College London, London SW7 2BX, UK; 7 Waters Research Center, 1031 Budapest, Hungary; 8 School of Computing, Queen’s University, Kingston, ON K7L 2N8, Canada; 9 Waters Corporation, Wilmslow SK9 4AX, UK

**Keywords:** REIMS, ambient ionization mass spectrometry, multi-site, reference material, food-grade meat

## Abstract

Rapid evaporative ionization mass spectrometry (REIMS) is a direct tissue metabolic profiling technique used to accurately classify tissues using pre-built mass spectral databases. The reproducibility of the analytical equipment, methodology and tissue classification algorithms has yet to be evaluated over multiple sites, which is an essential step for developing this technique for future clinical applications. In this study, we harmonized REIMS methodology using single-source reference material across four sites with identical equipment: Imperial College London (UK); Waters Research Centre (Hungary); Maastricht University (The Netherlands); and Queen’s University (Canada). We observed that method harmonization resulted in reduced spectral variability across sites. Each site then analyzed four different types of locally-sourced food-grade animal tissue. Tissue recognition models were created at each site using multivariate statistical analysis based on the different metabolic profiles observed in the *m/z* range of 600–1000, and these models were tested against data obtained at the other sites. Cross-validation by site resulted in 100% correct classification of two reference tissues and 69–100% correct classification for food-grade meat samples. While we were able to successfully minimize between-site variability in REIMS signals, differences in animal tissue from local sources led to significant variability in the accuracy of an individual site’s model. Our results inform future multi-site REIMS studies applied to clinical samples and emphasize the importance of carefully-annotated samples that encompass sufficient population diversity.

## 1. Introduction

**Figure 1:**
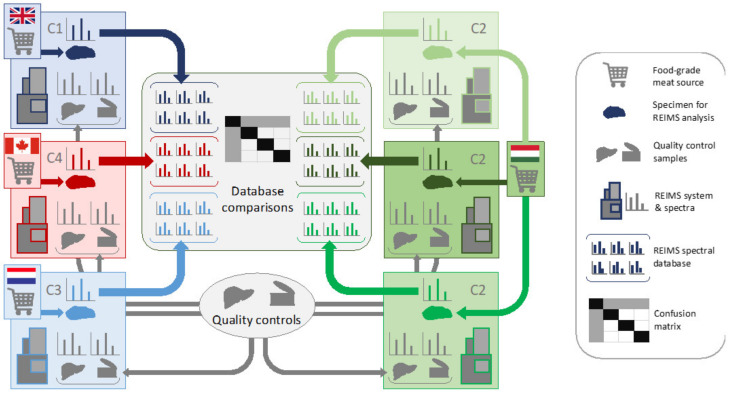
Multicenter ring trial for testing of REIMS methodology. Four consortium sites comprised: Imperial College London (Center 1, C1); Waters Research Center, Budapest, Hungary (C2); Maastricht MultiModal Molecular Imaging Institute, Maastricht The Netherlands (C3); and Queen’s University, Kingston, Canada (C4). Two single-source reference materials and four types of local food-grade meat were analyzed by the four centers, and classification models were based on a single center. Data was tested against data from the other centers. Reference material analyzed by all four centers comprised two batches of pork liver and meat homogenate (National Institute of Standards and Technology (NIST), Standard reference material 1546a). Local sources of food-grade meat included calf liver, chicken liver, chicken breast and turkey breast. Three different instruments were used to analyze the food grade meat at C2.

Rapid evaporative ionization mass spectrometry (REIMS) has opened up the possibility of near real-time sampling and identification of biospecimens, where classification is based on multivariate statistical analysis using mass spectra from pre-built mass spectral databases [1]. During REIMS, gas-phase ions from major tissue components are produced by various surgical energy devices. Unlike other mass spectrometry-based techniques used for sampling tissue, such as mass spectrometry imaging [2], REIMS does not require time-consuming sample preparation, enabling direct sampling of unprocessed solid tissue in situ, including vital tissue during surgery [3]. Analysis of a broad range of sample types by REIMS has been reported in the literature, including cultured cell lines [4,5], microorganisms [6,7,8] and food samples [9,10,11,12]. Considerable attention has been given to the ability of REIMS to differentiate malignant from normal tissue types associated with human breast [13], colon [14], ovary [15] and cervix [16], emphasizing its potential as an intraoperative pathology tool during cancer surgery. The sensitivity and specificity of tumor tissue detection reported in these studies ranged from 88.5–100%, partly due to the high prevalence of MS peaks corresponding to glycerophospholipids whose metabolism is altered in malignant tissue [17]. A general observation from all of these studies is that, while most of the same chemical species can be detected across tissue types and pathologies, it is the relative abundance of its constituents that is specific to a given tissue type as opposed to the presence or absence of individual biomarkers, with the exception of infectious diseases. A potential limitation of REIMS-based tissue classification is its reliance on complex chemical profiles, where absolute quantification of individual species is not straightforward. Most studies were conducted at a single site with 1–2 instruments, thus the inter-site and inter-instrument reproducibility and variability has not been assessed to date. In order for REIMS to achieve its full potential, a multi-site study of the reproducibility of REIMS analytical workflow and classification algorithms needs to be conducted. We set out to: (1) create a consortium of laboratories with identical REIMS equipment; (2) identify single-source reference material appropriate for multi-site REIMS testing; (3) harmonize data acquisition and processing methodology; and (4) test the methodology and tissue classification accuracy on single-source reference material and local food-grade meat (Figure 1). Lessons learned from the current study are anticipated to inform future multi-site REIMS analyses on clinically-relevant tissue samples.

## 2. Materials and Methods

### 2.1. Samples and Logistics

Reference samples consisted of: (1) NIST reference meat homogenate (Standard reference material^®^ 1546a, National Institute of Standards and Technology, NIST); (2) two individual batches of pork liver procured by Imperial College London (Center 1 (C1), London, UK; and (3) test samples from four types of food-grade meat samples (calf liver, chicken liver, chicken breast, turkey breast) procured from local supermarkets analyzed at each center (Figure 1). All samples were stored at −80 °C after being received at each laboratory and prior to analysis.

For the porcine reference material, frozen (−80 °C) liver samples were acquired by C1 from Fresh Tissue Supplies (East Sussex, UK). When the specimens were received, they were immediately stored at −80 °C. For batch 1, a dedicated and homogeneous liver was used (from a single animal), and after it was left to thaw at room temperature, multiple samples were cut to the approximate size of 0.5 × 2 cm and stored in individual 2-mL vials for further storage and distribution. During dissection, samples were taken from both right and left lobes. Samples containing residual hepatic duct/arteries/veins were excluded. Samples near the gallbladder and cystic ducts were also excluded. For batch 2, the same process was followed with a different piece of liver from another animal. After processing, the samples were shipped on dry ice to the other three participating centers: Waters Research Center, Budapest, Hungary (C2); Maastricht MultiModal Molecular Imaging Institute, Maastricht, The Netherlands (C3); and Queen’s University, Kingston, Canada (C4).

### 2.2. Sampling with Diathermic Knife

Tissue samples were allowed to reach room temperature before analysis and placed on an electrosurgical return electrode. If necessary, samples were wetted with deionized water. Electrosurgical dissection was carried out using commercial electrosurgical generators (C1: COVIDIEN Ltd. Triad, Dublin, Ireland; C2: ERBE Elektromedizin GmbH ICC-350, Tübingen, Germany; C3/C4: COVIDIEN Ltd. Force Fx, Dublin, Ireland) providing power-controlled sinusoidal at 330 kHz alternating current. Tissue was sampled using a custom diathermy handpiece with an aerosol extraction line (Waters Research Center, Budapest, Hungary) [1]. Following a common protocol, the generator was used in cut mode with an optimized power setting, i.e., the lowest power setting capable of continuously cutting the sample (10 W for the NIST reference meat homogenate and 20 W for the pork liver and food-grade tissue samples). In order to maximize reproducibility, the diathermy electrode was positioned at a 40° angle above the tissue during sampling. NIST meat homogenate and pork liver reference samples were used to assess instrument variability across sites. Data acquisition was performed two times per day (morning and afternoon) on two consecutive days for each sample. Each measurement consisted of 3–5 burns, typically lasting 4–8 s in duration.

### 2.3. REIMS—Q-TOF Instrumentation

At each site, data were acquired on a Xevo G2-XS quadrupole time-of-flight (Q-TOF) mass spectrometer fitted with a REIMS source (version III) (Waters, Wilmslow, UK). Operating parameters were kept constant among sites, and instrument status was checked using a common checklist (Supplementary Figure S1). Instruments were connected to a 7 bar gas (pressurized air or Nitrogen) supply. TOF and backing pressures were in the range of <10^−7^ and 1.3 mbar, respectively. The aerosol produced by electrosurgery was aspirated via a Venturi air jet pump connected to the REIMS interface. The heated coil in the REIMS source was kept at 800–900 °C. Data were acquired in “sensitivity” and negative ionization mode within the *m/z* range of 100–1500. The mass resolution was above 15,000 full width at half-maximum. Instrument calibration was performed or checked using sodium formate before each measurement series. A solution of leucine–enkephalin (LeuEnk) (Sigma-Aldrich, Gillingham, UK) at a concentration of 0.05 ng/µL (prepared in UHPLC-grade isopropanol, VWR) was continuously infused during acquisition at a flow rate of 150 µL/min for internal lock mass correction [18].

### 2.4. Data Analysis

Mass spectral processing and multivariate data analysis were performed using the Abstract Model Builder (AMX) software ([beta] version 1.0.1581.0, Waters Research Center). All mass spectra were processed as follows: (i) background-subtracted; (ii) lock mass corrected against the reference peak of deprotonated LeuEnk [M-H]^−^ at *m/z* 554.2615; (iii) binned to 0.1 Da (within the *m/z* range of 600–1000—corresponding to the region of abundant phospholipids and triglycerides); (iv) normalized against the total ion count (TIC). Multivariate analysis was based on principal component analysis/linear discriminant analysis (PCA/LDA). PCA was performed with a maximum of *n* = 25 dimensions and LDA with *n*−1 dimensions, where *n* corresponds to the number of classes introduced in the model. Cross-validation tests were performed by building the site-specific classifiers to classify the data generated at the other sites. Data points deviating five times of standard deviation (SD) were marked as outliers. In order to identify specific peaks differentially-abundant by site and by tissue type (i.e., to find the smallest subset of peaks accounting for the best separation of (1) each site (2) each tissue type); peak picking was done by using linear support vector comparison (LSVC) optimized for number of peaks <20 and achieving >95% correct classification and by selecting the top 12 ranked peaks of the first 3 loading plots for LDA. Plots of representative mass spectra were generated in MassLynx 4.1 by combining 6–7 scans across a burn, performing lock mass correction (as described above), background subtraction and peak centroiding. Peak lists from processed spectra were exported and plotted in GraphPad Prism v.9.

## 3. Results

### 3.1. Multi-Center Characterization of Pork Liver and NIST Meat Homogenate

**Figure 2:**
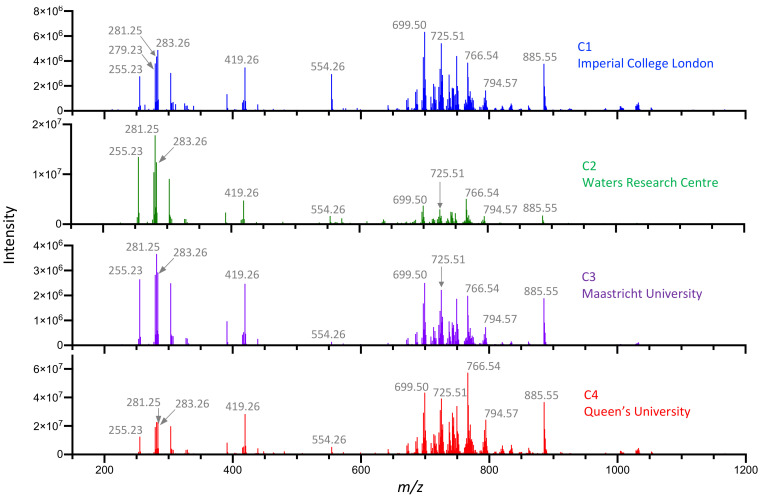
REIMS spectra of single-source pork liver acquired at each center. A single source of pork liver (Batch 1) was distributed by C1 to all other centers. REIMS spectra comparison of pork liver from each center in the *m/z* range of 100–1200. Each representative spectrum shown is the average of 6–7 individual scans.

Reference pork liver (Batch 1) was analyzed at each center (C1, C2, C3 and C4) using identical instruments. We compared mass spectra using LSVC and qualitative methods to identify features that differentiated spectra by center. Mass spectra comprised a range of fatty acids (FAs) and phospholipid species including phosphatidylethanolamines (PEs) and phosphatidylinositols (PIs), among others, as shown in Figure 2.

**Figure 3:**
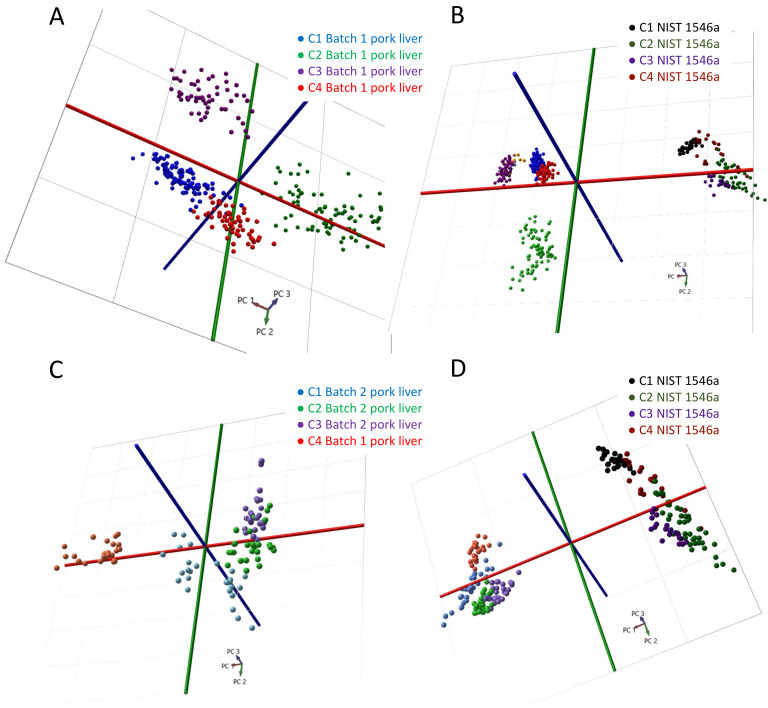
Multivariate analysis of REIMS spectra from reference pork liver and NIST meat homogenate determined at each center. PCA was used to study variability in multiple REIMS spectra acquired from a single source of pork liver before (variance explained by PC1, PC2 and PC3 are respectively 59.62%, 18.14% and 9.86%) (**A**) and after method harmonization (variance explained by PC1, PC2 and PC3 are respectively 63.83%, 10.85% and 8.03%) (**C**). PCA was also used to study differences in spectra among centers and two types of reference material (pork liver and NIST meat homogenate) prior to (variance explained by PC1, PC2 and PC3 are respectively 71.87%, 14.56% and 4.05%) (**B**) and after method harmonization (variance explained by PC1, PC2 and PC3 are respectively 34.1%, 21.03% and 12.49%) (**D**).

Most of the FA species observed included: palmitic acid *m/z* 255.23 [FA(16:0)-H]^−^; linoleic acid *m/z* 279.23 [FA(18:2)-H]^−^; oleic acid *m/z* 281.25 [FA(18:1)-H]^−^; stearic acid *m/z* 283.26 [FA(18:0)-H]^−^*;* and arachidonic acid *m/z* 303.23 [FA(20:4)-H]^−^. The major glycerophospholipid observed species included: *m/z* 699.50 [PA(36:2)-H]^−^ and [PE(34:1)-NH_4_]^−^; 725.51 [PE(36:2)-NH_4_]^−^; 742.54 [PE(36:2)-H]^−^; 766.54 [PE(38:4)-H]^−^; 865.58 [PI(36:0)-H]^−^; and *m/z* 885.55 [PI(38:4)-H]^−^, comprising mostly PAs, PIs and PEs, where *m/z* peak identities were previously confirmed by exact mass and tandem MS fragmentation of selected molecular ion species. Differences in the ratios between fatty acids and phospholipids were observed among sites, with C2 exhibiting the highest fatty acid-to-phospholipid ratio and C4 exhibiting the lowest (C2 > C3 > C1 > C4). More importantly, differences in ratios between certain phospholipids were apparent, within the *m/z* range we used for model creation and classification. For example, *m/z* 766.54 was the most abundant species in spectra acquired at C2, but *m/z* 699.50 was the most abundant in C1 and C3 spectra, based on the same piece of pork liver (Figure 2). We focused on the *m/z* range of 600–1000, as intact structural or storage lipid metabolites can be easily identified and can be utilized for robust tissue-type identification due to low background noise and minimal interferences. LSVC of pork liver spectra revealed a correct classification rate of 97% based on cross-validation by site, using only three peaks: *m/z* 699.50, 766.51 and 885.55 (Supplementary Figure S2A–C and Table S1). The 3D-PCA plot displayed in Figure 3A revealed center-dependent grouping of spectra based on the *m/z* 600–1000 range. We then plotted the spectra acquired from NIST meat homogenate alongside those from the batch 1 pork liver in the same *m/z* range (Figure 3B). This revealed distinct groups of spectra associated with either pork liver or NIST meat homogenate (alongside the PC2 axis) regardless of center, although center-dependent sub-groups of pork liver spectra were still clearly distinguishable.

To further minimize the center-dependent variability, we harmonized all instrument parameters and created a pre-acquisition checklist, completed by all sites prior to analysis (Figure S1). We then repeated the pork liver and NIST meat homogenate comparison experiments. Centers 1–3 used a second batch of pork liver (Batch 2); however, this sample was not available at C4, where batch 1 was used instead. From the PCA plot of the pork liver spectra in Figure 3C, it was evident that between-center variability was reduced significantly in comparison to the previous analysis (Figure 3A). It was concluded that the remaining center-to-center variability was insignificant because spectra from the meat homogenate and pork liver grouped into distinct clusters on PCA and the center-specific subgroups could not be easily discerned, even though a different batch of reference pork liver was used by C4 (Figure 3D). While optimized LSVC trained to separate the samples based on the center was still able to achieve 98% correct cross-validation by site using pork liver reference material, 13 peaks were required, as compared to 3 peaks prior to method harmonization. The relative intensity of *m/z* 885.55 was four times greater for C4, presumably due to use of the batch 1 pork liver reference (Figure S2D–F and Table S1).

To determine if there were any analyst-dependent differences in lipid profiles, seven analysts from each center conducted multiple REIMS sampling points on batch 1 pork liver using the same instrument at C3. None of the analysts were permitted to observe the sampling technique used by the other analysts, in order to avoid the introduction of sampling bias. PCA of at least three sampling points per analyst revealed no separation of profiles based on the individual analyst. This observation confirmed that slight differences in how REIMS burns were conducted by each center’s analyst were unlikely to be a source of variability in between-center comparison of profiles in subsequent experiments (Figure S3).

### 3.2. Multi-Site Classification of Local Food-Grade Meats

**Figure 4:**
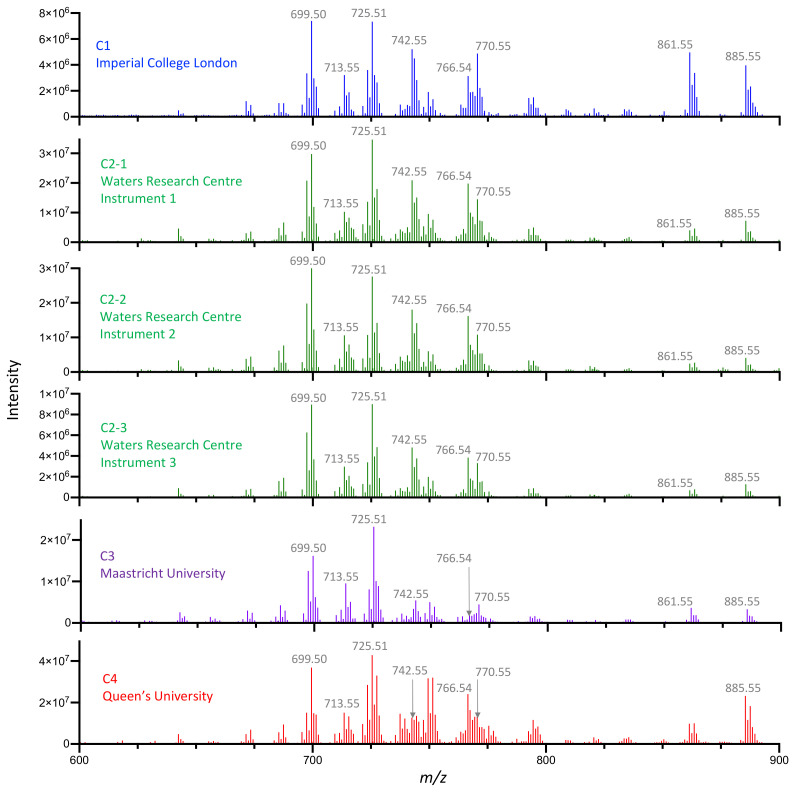
REIMS spectra of locally-sourced calf liver acquired at each center. Using harmonized analytical conditions, each site analyzed locally-sourced food-grade meats. Spectra from calf liver are presented, focusing on *m/z* range of 600–1000. For this study, C2 analyzed each sample on three different instruments. The representative spectra shown are the average of 6–7 individual scans.

**Figure 5:**
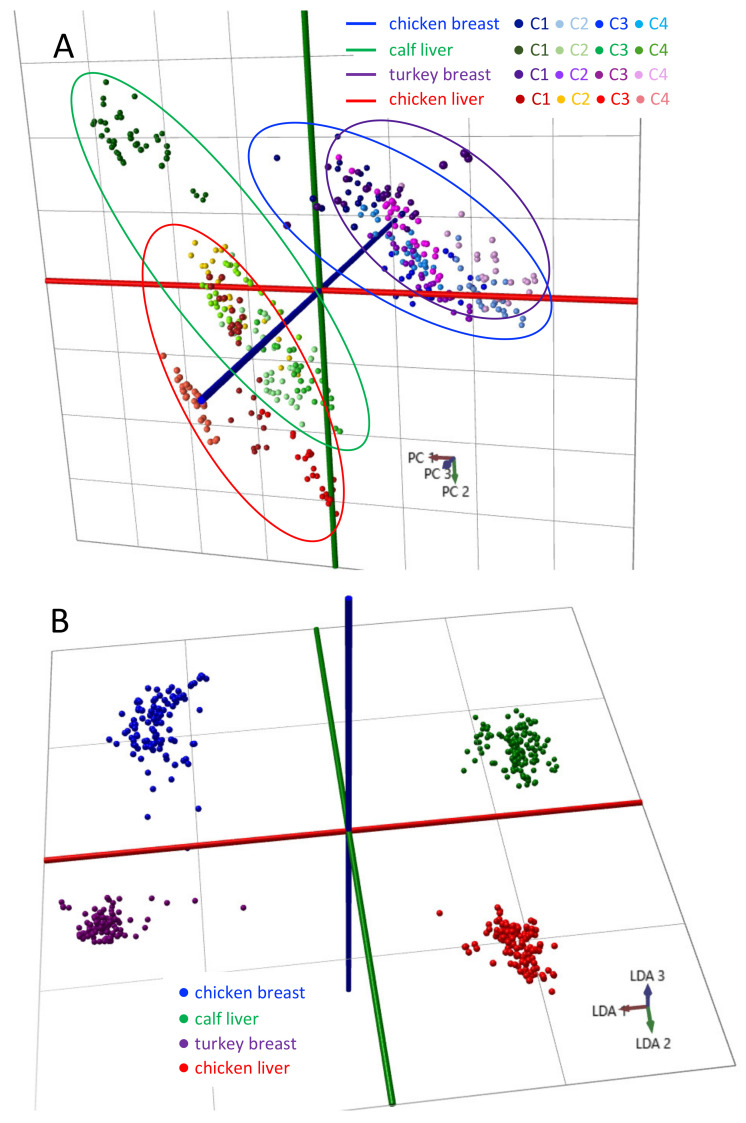
Multivariate analysis of REIMS spectra acquired from local sources of food-grade meat. Each center analyzed meat from local sources, including chicken breast, turkey breast, calf liver and chicken liver. The PCA (**A**) and PCA/LDA (**B**) plots compare the overall and tissue-type-based variability among sites.

Each center acquired four different types of food-grade animal tissue from local sources that were analyzed using our harmonized methodology. Sufficient sample was obtained at C2 to enable analysis over three different instruments (C2-1, C2-2, C2-3), whereas all other centers used a single instrument (Figure 1). A total of 2435 scans were selected from 487 data points comprising calf liver—126 data points (630 scans); chicken breast—105 data points (525 scans); chicken liver—147 data points (735 scans); turkey breast 109 data points (545 scans). The *m/z* 600–1000 range of spectra from locally-sourced calf liver across all instruments (Figure 4) revealed variability associated with different sources of calf liver, but no significant variability was apparent when analyzing the same calf liver across multiple instruments at C2. The multivariate plots in Figure 5 depict the distribution of all of the spectra using both PCA (Figure 5A) and PCA followed by an LDA (Figure 5B), which was used for model creation, classification and testing. The same characteristics were observed with the other tissue types across the sites.

Individual statistical models were created using data from each center, and the number of correct classifications were determined by testing these models against data from the remaining centers (Table 1). Correct classification rates ranged between 69–100%, where C2 achieved the best correct classification rate and C4 the worst, with models from C1 and C3 achieving correct classification rates of 75% and 88%. We note that the model for C2 was based on a larger number of spectra from three instruments and that C4 was the only non-European center. Given that cross-validation between three instruments at C2 using the same source of tissue was also 100%, we conclude that incorrect classifications likely occurred because of differences in sources rather than instrument-to-instrument variability, especially when creating a model with North American samples and testing it against European ones.

All incorrect classifications were based on calf liver being classified as chicken liver, and chicken breast being classified as turkey breast. When the pork liver reference samples were included in the analysis, each model 100% correctly classified the pork liver, confirming that between-instrument variability was insignificant, relative to the variability associated with center-specific sources of samples.

**Figure 6:**
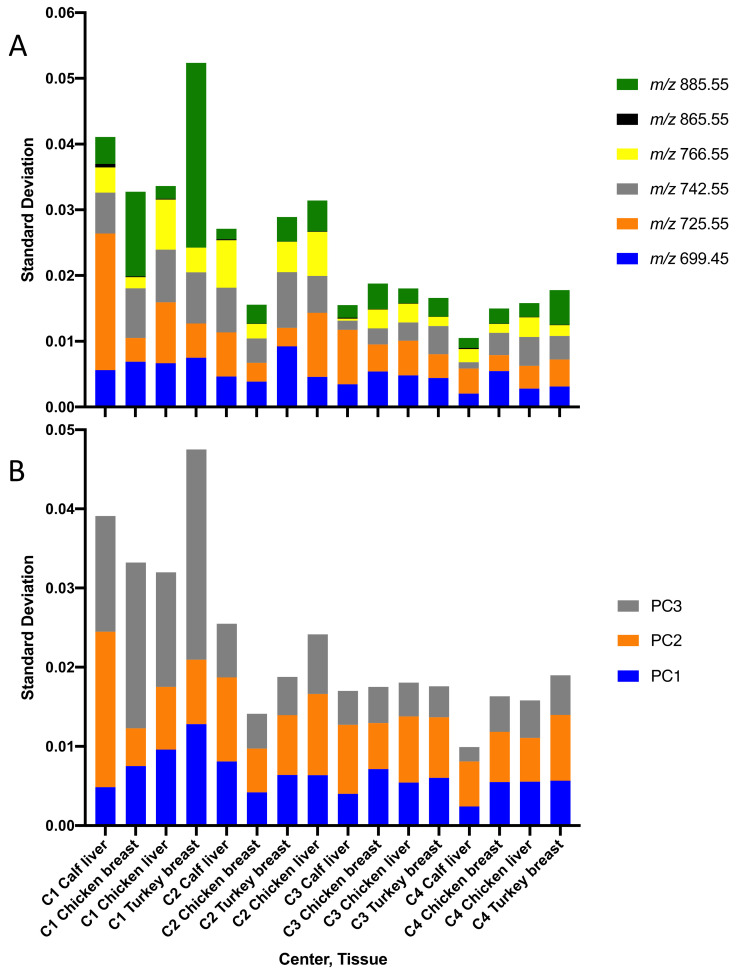
Standard deviation of selected phospholipid peaks and principal components. Assessment of variability in major phospholipid peaks (**A**) and principal components 1–3 for local food grade meats analyzed by REIMS at each center (**B**).

Tissue recognition model at C2 appeared to encompass the variability associated with heterogeneity within sample types at all centers, as the model exhibited a 100% correct classification rate. As the C2 model was generated using spectra acquired over three technically identical instruments, we presumed that a greater number of spectra over the three instruments was statistically advantageous. However, a model created from a single instrument at C2 also exhibited a near-perfect classification rate. Even though the variance of C1 was greater than C2 as determined by the standard deviation of the PCs 1–3 (Figure 6A) and major phospholipid peaks (Figure 6B), C1 exhibited a correct classification rate of only 88%, suggesting that variance was not the only factor that contributed to the success of the C2 model. Furthermore, while it can be rationalized that differences in North American meat (C4) versus European meat would place the C4 model at a clear disadvantage, this model also exhibited the lowest variance and accordingly the poorest correct classification rate. On the other hand, C4 spectra were well-classified by most other models. The variance of the C3 model was slightly greater than C4 and exhibited a significantly improved correct classification rate of 89% as compared with 70%. Taken together, the overall success of the C2 model was likely due to a combination of variance and its appearance to be the best representative or “average” source of samples compared to other sites.

Site-dependent peak picking was done by using LSVC and LDA loading plots as described above. Peaks found with both algorithms accounting for the difference between sites were removed from analysis, and cross-validation by site was performed by training on data acquired at one site and performing classification on data acquired at all other sites. Correct classification rates for C1–C3 models were 0–5% lower than those presented in Table 1, even when using the full spectrum instead of the *m/z* 600–1000 range. The model, trained on data from C4, improved marginally from 69.9–72.3% when top-ranked peaks were removed. It is worth noting that the most significant peaks accounting for the differences between sites (*n* = 46) and meats (*n* = 22) using LSVC (C = 50) had a significant overlap (*n* = 19), suggesting that there were no site-specific different peaks appearing in the signal; rather there was a difference in the fingerprint. We continued our analysis on a subset of the peaks with C = 5 parameter as the 4-fold cross-validation resulted in a 100% correct classification rate. Peaks are shown in Table S1. Similarly, when LSVC was optimized to achieve >95% correct cross-validation by tissue type or site, 10/19 peaks associated with type overlapped with site (Table S1, Figures S4 and S5). When leave-one-site-out cross-validation was performed with 19 peaks, the overall correct classification of the meat was 84.8% as compared to 87.7%, using all peaks within the *m*/*z* 600–1000 range.

Given the considerable overlap of spectra between samples of the same tissue type (muscle, liver), regardless of species on PCA, it was not surprising that all incorrect classifications were due to the inability to differentiate between species (chicken or calf) of the same type of tissue, whereas models always correctly classified the tissue type (muscle/breast vs. liver). Among the peaks selected by LSVC to optimally differentiate the food grade tissues, relative concentrations appeared to trend with the tissue type. For example, *m/z* 766.55 was elevated in both chicken liver and calf liver, relative to both chicken breast and turkey breast (Figure S4). Taken together, these findings strengthen our theory that the composition of different samples in different countries accounts for slight variability in the spectral fingerprint that can sometimes obscure the correct identification of species, but that is unrelated to differences in mass spectrometry equipment or set-up.

## 4. Discussion

We formed an international consortium to evaluate the robustness of the REIMS technology across four sites and tested multivariate models created at each site to evaluate the potential clinical utility of REIMS. Of importance was the circulation of single-source reference material that was analyzed by all sites. While “batch 2” pork liver was not received by C4 owing to challenges associated with the importation of food-grade meat for scientific research, all centers successfully received batch 1 pork liver and were able to procure the NIST meat homogenate. Using identical equipment, our findings demonstrated that variability in REIMS spectra can be minimized by harmonizing sampling methods and instrument settings. Despite some inevitable variability in spectral profiles across sites, classification accuracy was 100% for the reference material when each site’s model was tested against other sites’ data, suggesting that the level of analytical variability achieved was sufficient. The range of correct classification rates (69–100%) by site-specific models for food-grade meats points to other sources of between-site variability such as: (1) relative variance in testing versus validation datasets and (2) differences in local sources of the same type of meat, such as animal breed, leading to differences in the composition of major phospholipid peaks. Despite the lower performance of some models owing to misclassification of animal species, the tissue-type classification was correct 100% of the time.

The importance of variance in PCA/LDA-based classifiers of tissue was demonstrated in a study that used both diathermy (CV = 14–23%) and CO_2_ laser (CV 9–12%) to analyze an identical series of tissue samples [19]. Using a diathermy-based model (larger variance) to classify the laser data gave an accuracy of 58%. Conversely, a model based on the more precise laser data tested against diathermy halved the accuracy, but the accuracy could be restored to 58% if tissue-specific peak lists were used for model creation and testing [19]. This work suggests that the impact of variance on misclassifications could be minimized by using a more targeted approach. Among our study’s diathermy-based models, the combination of a more precise model and being the only non-EU meat source may have contributed to the poor accuracy of C4′s model, even though profiles acquired at C4 were accurately classified by other models. Gredell et al. used a combination of dimension-reduction and classification algorithms to study REIMS profiles across different quality measures of food-grade beef, such as muscle tenderness and breed [20]. The group found that the specific algorithm type, which gave the best classification accuracy, varied by quality measure. This study suggests that the impact of site-specific sources of variability could be optimized by certain algorithms. Evaluation of additional algorithms and also different instrument types may serve as important future directions.

The range of classification accuracies for food-grade meat samples among models was anticipated, given the range of animal husbandry practices across regions; potential variation in meat processing practices; and lack of international harmonization on the use of growth promoters. A preliminary study of REIMS-based sampling of lamb loin from New Zealand, England and Wales revealed region-specific clusters using PCA [21]. Importantly, the report cautioned that apparent differences may be due to multiple factors, including the specific breed of animal, feeding practices and slaughtering methods. In studies aimed at investigating more specific differences in food-grade meat, REIMS has been shown to differentiate between organic and conventional beef across tissue types (84% accuracy) [22] and between dry-aged beef and beef subjected to other aging practices [23]. Furthermore, >95% classification accuracy was observed in differentiating pork from animals given the growth promoter ractopamine and untreated animals across loin, shoulder and thigh tissue [11]. He et al. also demonstrated high accuracy in predicting whether beef muscle was freeze-thawed, based on patterns of phosphatidic acid and fatty acid composition discerned from REIMS spectra [24]. Our site-specific models for food-grade meat were based on single sources of meat and, thus, only accounted for minimal diversity in animal husbandry and processing practices known to affect REIMS profiles. Furthermore, we were unable to trace the specific breeds of animal tissue used. Controlling for all these parameters in single sources of meat would have proven challenging at the retail level and would have necessitated sampling across multiple sources. Despite this limitation, the type of meat tissue was always correctly classified across sites.

Use of food-grade meat purchased from local grocery stores aimed to introduce variability in meat sources that could not be easily controlled, as a way to parallel the inevitable tissue variability associated with clinical samples. Given that our goal is to apply the current methodology across sites for analysis of clinical samples, our study points to the importance of well-annotated samples using gold-standard methods (such as histopathology) and a sufficient number of samples that encompasses population diversity. Clinical tissue classification models will be required to accurately differentiate relevant tissue types at multiple sites regardless of environmental and certain genetic factors that may contribute to tissue heterogeneity. Harmonized analytical and sample handling methodology are important steps towards this goal.

## Figures and Tables

**Table 1:** Correct classification rate for all types of food-grade tissue using models created at individual sites.

Model Used for Training ↓	Correct Classification Rate (%)						
	Total	C1	C2-1	C2-2	C2-3	C3	C4
C1	88.24	-	71.43	100.0	75.0	93.1	100.0
C2 (all) ^1^	100.0	100.0	-	-	-	100.0	100.0
C2-1	98.17	98.2	-	100.0	100.0	97.0	98.2
C3	88.9	100.0	76.19	50.0	85.7	-	100.0
C4	69.9	83.2	59.0	50.0	50.0	77.2	-

^1^ denotes model created from all three instruments at C2.

## Data Availability

Data available in manuscript and Supplementary Material.

## Supplementary Materials

The following supporting information can be downloaded at: https://www.mdpi.com/article/10.3390/metabo12111130/s1, Figure S1: REIMS system set-up and suitability checklist; Figure S2: Relative abundance of selected *m/z* bins in pork liver reference material by center; Figure S3: Analyst-dependent differences in REIMS spectra; Figure S4: Relative abundance of selected *m/z* bins in food-grade meats by meat type; Figure S5: Relative abundance of selected *m/z* bins in food-grade meats by center; Table S1: LSVC analysis of pork liver reference material and food-grade meats.
