# Supplementary material for: Harmonization of Rapid Evaporative Ionization Mass Spectrometry Workflows across Four Sites and Testing Using Reference Material and Local Food-Grade Meats
Source: Metabolites. 2022 Nov 17;12(11):1130. doi: 10.3390/metabo12111130 (PMC9699633; doi:10.3390/metabo12111130)
Supplement: Supplementary file 1 [file metabolites-12-01130-s001.zip › metabolites-1982512-supplementary.pdf]

__Supplementary Material__

Harmonization of Rapid Evaporative Ionization Mass Spectrometry Workflows across Four Sites and Testing Using Reference Material and Local Food-Grade Meats

Martin Kaufmann[1,2‡], Pierre-Maxence Vaysse,[3,4,5‡] Adele Savage,[6] Ala Amgheib,[6] András Marton,[7] Eftychios Manoli[6], Gabor Fichtinger,[8] Steven D. Pringle,[9] John F. Rudan,[1] Ron M.A. Heeren,[3] Zoltán Takáts,[6] Júlia Balog,[6,7]* Tiffany Porta Siegel[3]*

[1] Department of Surgery, Queen's University, Kingston, ON K7L 2V7, Canada
[2] Department of Biomedical and Molecular Sciences, Queen's University, Kingston, ON K7L 3N6, Canada
[3] Maastricht MultiModal Molecular Imaging (M4I) Institute, Division of Imaging Mass Spectrometry, Maastricht University, 6229 ER Maastricht, The Netherlands
[4] Department of Surgery, Maastricht University Medical Center + (MUMC+), 6229 HX Maastricht, The Netherlands
[5] Department of Otorhinolaryngology, Head & Neck Surgery, MUMC$^+$, 6229 HX Maastricht, The Netherlands
[6] Division of Computational and Systems Medicine, Department of Surgery and Cancer, Imperial College London, London SW7 2BX, UK
[7] Waters Research Center, 1031 Budapest, Hungary
[8] School of Computing, Queen's University, Kingston, ON K7L 2N8, Canada
[9] Waters Corporation, Wilmslow SK9 4AX, UK

2

Contents: 4

Figure S1: REIMS system set-up and suitability checklist 5

Figure S2: Relative abundance of selected *m/z* bins in pork liver reference material by center 6

Figure S3: Analyst-dependent differences in REIMS spectra 7

Figure S4: Relative abundance of selected *m/z* bins in food-grade meats by meat type 8

Figure S5: Relative abundance of selected *m/z* bins in food-grade meats by center

Table S1: LSVC analysis of pork liver reference material and food-grade meats

Supplementary Figure S1: REIMS System set-up and suitability checklist.

1. General

- ☐ Make sure the instrument has been in operate mode for 30 minutes
- ☐ If there is no signal on the instrument when solvent is flowing, try the following in the given order, and check if there is signal after each step:
  - o Reinitialise
  - o Change to other polarity mode (+) and back
  - o Put MassLynx into instrument standby (red), then switch to operate again
  - o Restart electronics

Instrument Settings:

- ☐ The correct .ipr file is loaded
- ☐ Negative ionzation mode is selected
- ☐ Sensitivity mode is selected
- ☐ Collision gas is ON
- ☐ Collision Energy is OFF
- ☐ Correct scan time is set (1s)
- ☐ Backing pressure is >1.5
- ☐ Collision pressure is between $1.5*10^{-1} – 2.5*10^{-1}$
- ☐ TOF pressure is below $1.1*10^{-6}$, but ideally below $9*10^{-7}$
- ☐ Check the following instrument parameters are set, when MS Profile Type is set to Auto P, the grey values don't matter anymore:

Sampling Cone	40	REIMS tab
Source Offset	80	REIMS tab
Collision RF Offset	150	Setup/RF settings
Collision RF Initial	50	
Collision RF Final	150	
MS Profile Type	Auto P	Setup/Quad profile
MSProfileMass1	100	
MSProfileDwellTime1	20	
MSProfileRampTime1	20	
MSProfileMass2	300	
MSProfileDwellTime2	20	

2. REIMS source parameters:

- [ ] Heater is turned ON
- [ ] Small IPA capillary is in place
- [ ] The impactor holder assembly is open
- [ ] After starting leu-enk in IPA, leu-enk peak is visible around 554.2
- [ ] There is no peak splitting of 554.2
- [ ] Check the resolution is >15000 (for this use ResCalc software)
- [ ] Correct mass range is set (usually 50-1200)
- [ ] Check if the file duration is set to 0 when starting a file

Routine REIMS checklist:

- [ ] The instrument has been calibrated/calibration was checked that day
- [ ] The instrument has been calibrated since last electronics shutdown/reboot
- [ ] The detector was calibrated in the past month

***Ex-vivo* breast tissue checklist:**

- [ ] The overall TIC (during burns) > 5e^7
- [ ] Leu-enk intensity level per scan 1e^5 - 5e^6 at the beginning
- [ ] Leu-enk intensity level per scan 1e^5 - 5e^6 at the end
- [ ] Tissue intensity level per scan (highest peak in the *m/z* 600-1000 range) > 5e^4
- [ ] Resolution > 15000 at the beginning of analysis
- [ ] Resolution > 15000 at the end of analysis

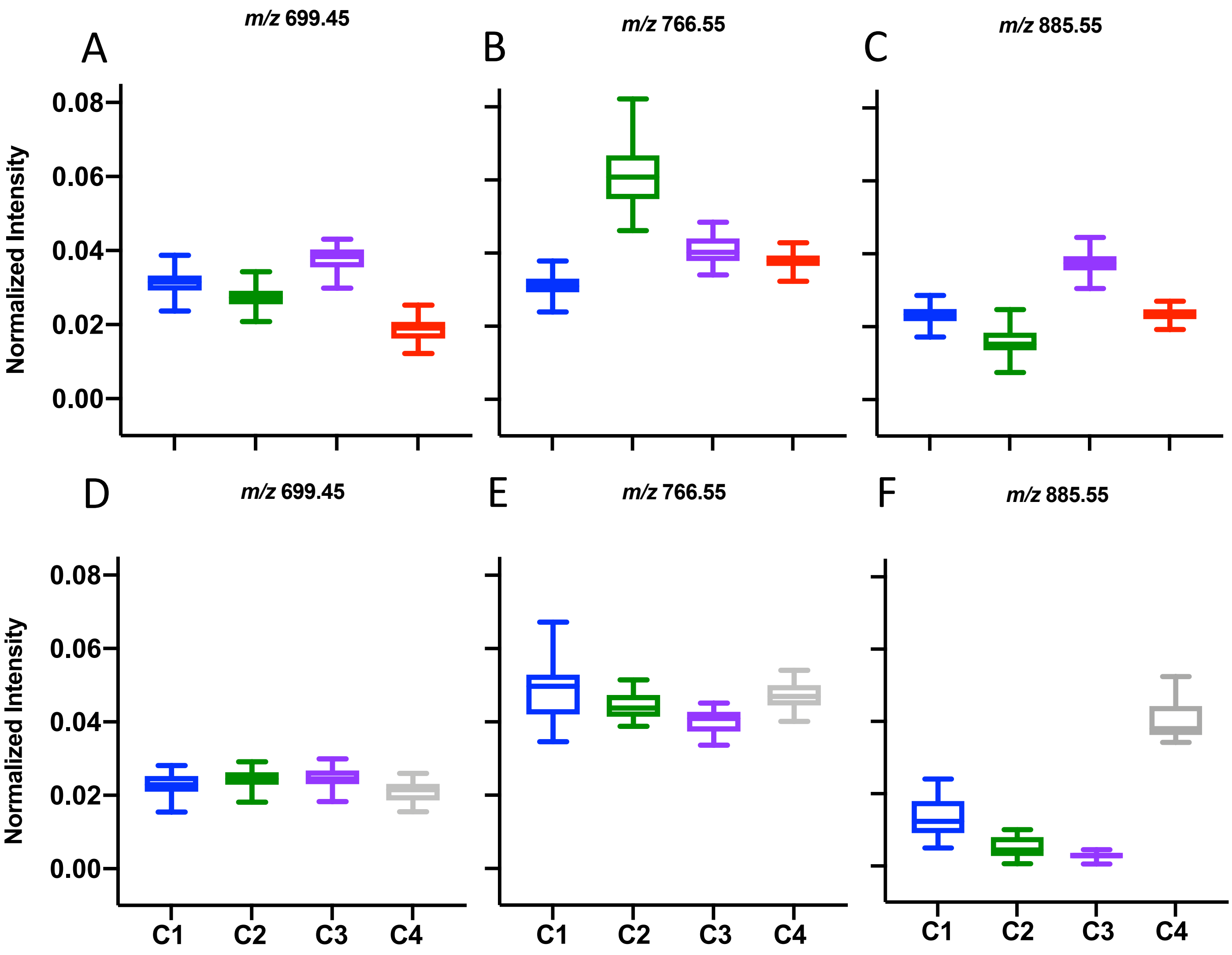

Supplementary Figure S2: Relative abundance of selected *m/z* bins in pork liver reference material by center. LSVC was used to identify *m/z* bins that correctly classified center >95% of the time (Table S1). Panels A-C present boxplots of 3/3 bins that achieved optimal cross-validation prior to method harmonization. Panels D-F show boxplots of the same *m/z* bins after method harmonization, which are a subset of the 13 *m/z* bins required to achieve optimal cross-validation by site, after method harmonization. C4 used used batch 1 pork liver for all analyses. C1-C3 used batch 1 pork liver prior to method harmonization, and batch 2 after method harmonization. P<0.0001 for all plots by non-parametric ANOVA.

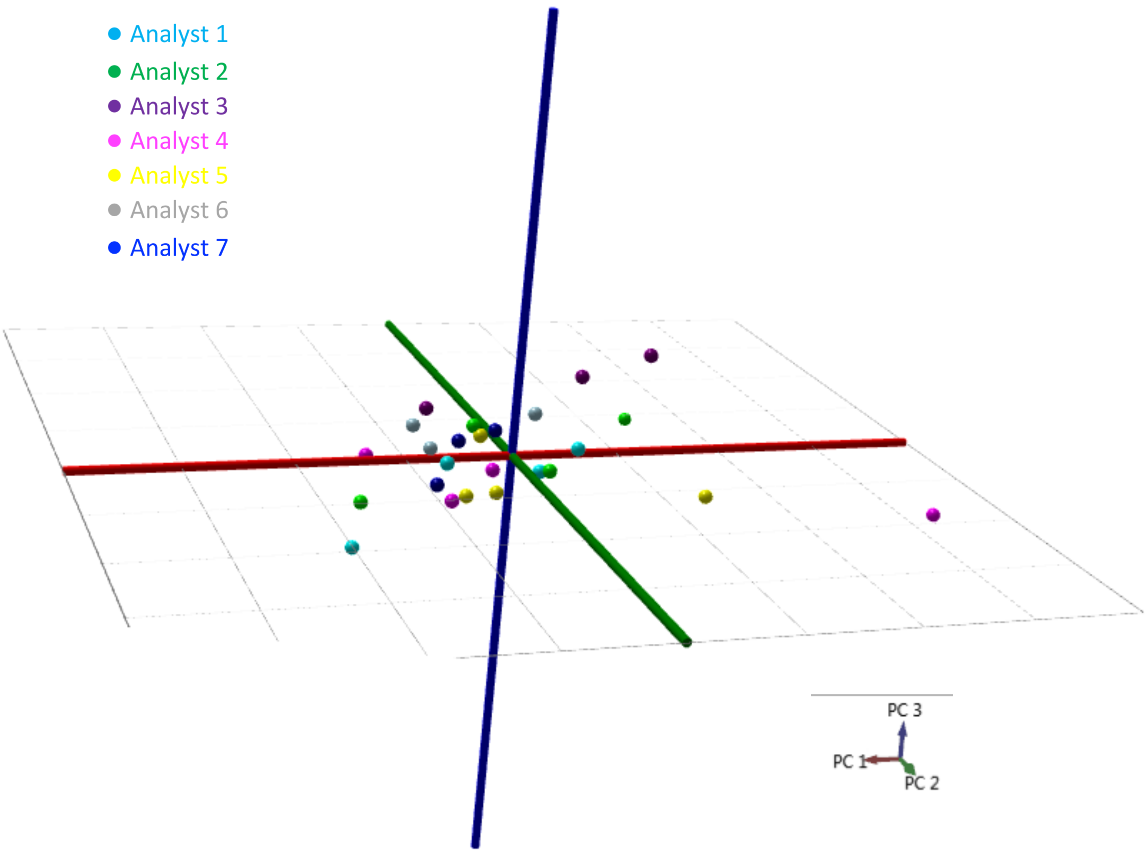

Supplementary Figure S3: Analyst-dependent differences in REIMS spectra. Seven analysts from all four centers conducted at least 3 REIMS analyses on batch 1 pork liver at C3. The PCA plot of the *m/z* 600-1000 region of the mass spectra revealed insignificant analyst-dependent differences associated with sampling technique.

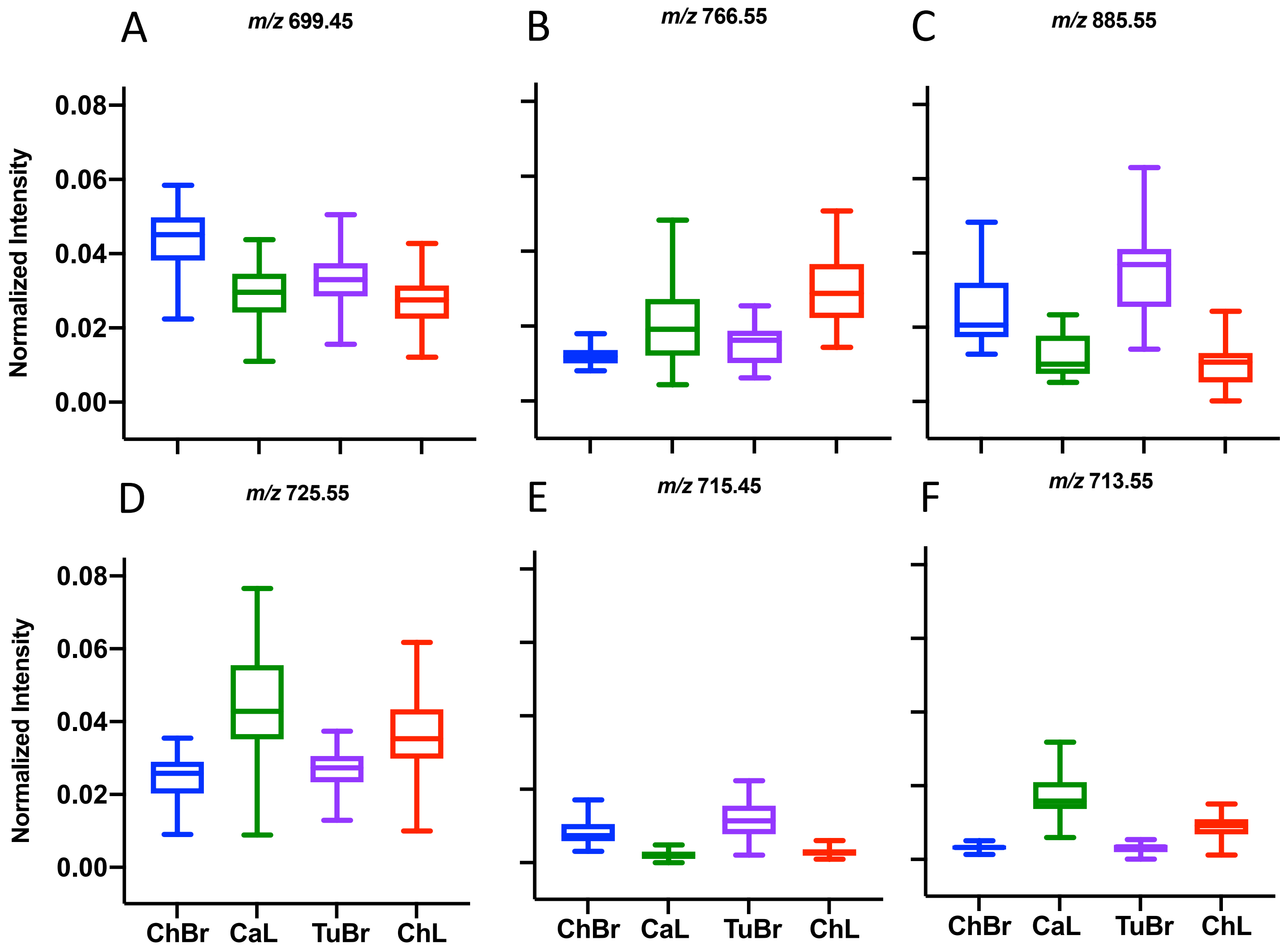

Supplementary Figure S4: Relative abundance of selected *m/z* bins in food-grade meats by meat type. LSVC was used to identify *m/z* bins that correctly classified center >95% of the time (Table S1). Panels A-F present boxplots of 6/14 bins that achieved optimal cross-validation. P<0.0001 for all plots by non-parametric ANOVA. ChBr: Chicken breast. CaL: Calf liver. TuBr: Turkey breast. ChL: Chicken liver..

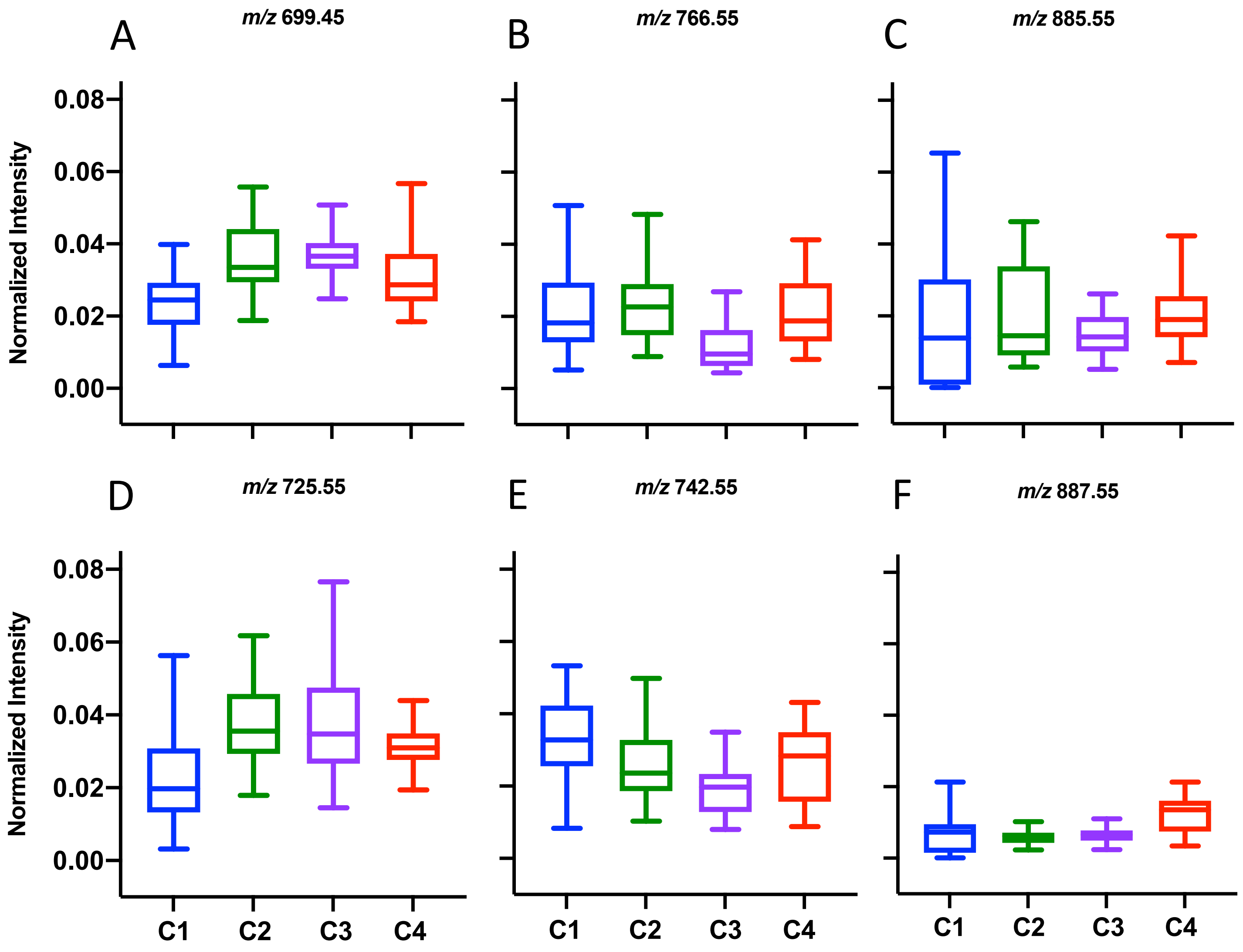

Supplementary Figure S5: Relative abundance of selected *m/z* bins in food-grade meats by center. LSVC was used to identify *m/z* bins that correctly classified all food-grade meats center >95% of the time (Table S1). Panels A-F present boxplots of 6/23 bins that achieved optimal cross-validation. $P<0.0001$ for all plots by non-parametric ANOVA.

Supplementary Table S1: LSVC analysis of pork liver reference material and food-grade meats

Analysis/Parameter	Pork Liver Ref.[1]	Pork Liver Ref.[1]	Food-grade meat[2]	Food-grade meat[2]
**Class for cross-validation**	Center	Center	Meat type	Center
**Correct classification rate**	97%	98%	84.8%	35%
**No. of mass bins**	3	13	19	20
**Selected *m/z* bin**				642.48
				671.47
				673.48
		682.59		
		695.46		
		697.48	697.48	697.48
	699.5	699.55	699.5	
			700.5	700.5
			705.48	705.48
			713.51	
			715.5	715.5
			721.48	
		723.5	723.5	
		725.51	725.51	725.51
			727.53	
				737.51
				738.51
		742.54		742.54
			743.53	743.53
			744.55	744.55
				747.5
			751.52	751.52
	766.54	766.54	766.54	766.54
		767.54		
			768.55	
		769.53		769.53
			770.57	
			773.53	773.53
		787.48		
		794.57		
				861.55
			863.56	
	885.55	885.55	885.55	
				887.56

[1]LSVC analysis optimized to achieve >95% correct classification rate for indicated samples and class, based on 4-fold cross-validation. Selected *m/z* bins are indicated.

[2] Standard LSVC and cross-validation by leaving one site out (meat type as class) or leaving one meat type out (center as class) results for food-grade meat by meat type or center. Selected *m/z* bins are indicated.
